# Feasibility of online group stress management training compared to web-based individual training for employees—a randomized pilot study

**DOI:** 10.3389/fpsyg.2025.1524285

**Published:** 2025-04-24

**Authors:** Leif Boß, Sandy Hannibal, Dirk Lehr

**Affiliations:** Department of Health Psychology and Applied Biological Psychology, Institute of Sustainability Psychology (ISP), Leuphana University of Lüneburg, Lüneburg, Germany

**Keywords:** internet-based interventions, videoconferencing, occupational stress, prevention, health promotion, patient preference, acceptance

## Abstract

**Background:**

In recent decades, digital stress management training, typically targeted at individuals, has gained increasing attention in health promotion. While these interventions show on average moderate to high effects on stress and other mental health outcomes, their use and acceptance in practice are often low. In contrast, group training may have advantages over these shortcomings. However, despite its widespread use in traditional non-digital health promotion, there is little evidence for digital training delivered in groups.

**Objective:**

This study’s aim was to explore the feasibility of live, online stress management training delivered in a group format and compare it to Internet-based training targeting individuals.

**Methods:**

Employees (*N* = 62), recruited from an open access website, were randomized into either group or individual training. Group training consisted of seven weekly online appointments led by a trainer and conducted via videoconference. Individual training consisted of seven web-based sessions which included written feedback provided by an e-coach after each session. The primary outcome was perceived stress eight weeks after training initiation. Feasibility was analyzed in terms of participants’ satisfaction, adherence, and perceived benefits of both training formats, assessed via both written questionnaires and interviews.

**Results:**

Participants in group training [Cohen’s d = 0.9 (95% confidence interval: 0.4 to 1.5)] and individual training [1.3 (0.6 to 2.0)] both experienced statistically-significant reductions in stress, with no significant difference between the two training formats [0.25 (−0.32 to 0.83); *p* = 0.579]. Full adherence rates were 70% in the group training and 50% in the individual training. Participants were satisfied with both formats, appreciating the social support and personal contact of the group setting, while appreciating the time flexibility and personal contact with an e-coach offered through individual training.

**Conclusion:**

This pilot study showed promising effects for the acceptance and health-related effectiveness of stress management training delivered in a group setting via videoconference. The findings highlight the value of personal contact with a coach and peers for positive user experiences during digital stress management interventions.

**Clinical trial registration:**

https://drks.de/search/en/trial/DRKS00024965, DRKS00024965.

## 1 Introduction

One major aim of occupational mental health promotion is to provide interventions to employees that can effectively reduce stress and prevent stress-related conditions such as depression, anxiety, or poor sleep. Meta-analyses have demonstrated that stress management training (SMT) is effective at reducing stress and improving general mental health (Richardson and Rothstein, 2008), and at reducing symptoms of depression and anxiety (Bhui et al., 2012; Martin et al., 2009; Tan et al., 2014).

Traditionally, stress management training is conducted in a group format and on-site (GroupSMT). While the contribution of GroupSMT programs to occupational health promotion is largely undisputed, their core characteristics have implications for implementation (Lehr et al., 2016). First, participation is limited to the dates and time slots offered, making it challenging for individuals with irregular working hours to attend meetings. Second, participation in a GroupSMT is not entirely self-determined, since the individual relies on enough others also signing up for the training course. Otherwise, the training sessions will not take place. Third, the location of the GroupSMT must be within a convenient distance to avoid time- and cost-consuming travel.

### 1.1 Digital stress management interventions

Digital interventions either targeting individuals or groups may complement traditional SMT. Over the past decade, internet-based SMT programs have gained increasing attention, typically targeting individuals (Individual-iSMT) (Heber et al., 2017). Such Individual-iSMT programs may overcome some of the obstacles of traditional SMT, while offering similar benefits for mental health (Stratton et al., 2021). For example, individuals can start the iSMT immediately; sessions are available at any time and accessible from any place; and participants can work through the intervention at their own pace. Meta-analyses have shown that iSMT programs are effective at reducing stress and depressive symptoms in different occupational groups, their effects increased when complemented by personal guidance (Phillips et al., 2019).

Digital stress management training can also be delivered in a group format (Group-iSMT). To date, evidence for the feasibility and efficacy of Group-iSMT programs in occupational health care is scarce (Phillips et al., 2019). In an early study, Wolever et al. (2012) piloted a mindfulness-based stress management training course in both a conventional in-person classroom and an online classroom with both formats reduced stress. In a retrospective analysis of a non-randomized study, Lim et al. (2021) compared the effects of group-based mindfulness training delivered on-site with an online version, and found that both versions reduced stress.

Individual and group iSMT programs each have potential advantages. Training in a group seems more attractive for employees than Individual-iSMT programs that provide no options for face-to-face contact with others (Apolinário-Hagen et al., 2020). From a theoretical point of view, Group-iSMT programs could offer additional advantages over Individual-iSMT. Stress management training can be described as a complex intervention (Craig et al., 2013), since such courses often encompass different coping methods and are delivered spanning numerous sessions over a period of several weeks (Lehr et al., 2016). According to Media Richness Theory (Daft and Lengel, 1984), the more complex and ambiguous the training content, the more extensive the media platform must be to minimize any potential misunderstanding or ambiguity of content. Thus, Group-iSMT may be beneficial relative to Individual-iSMT training, because participants and trainers can see and use the verbal and non-verbal expressions of each other in their communication. On the other hand, trainers must synchronously share their attention with several participants and adhere to the course process, whereas trainers (i.e., e-coaches) in Individual-iSMT programs can focus all their attention on a single person, especially when the coaching takes place through asynchronous, written feedback. Studies about the comparative communication effectiveness have, to date, revealed no differences between digital text-based and non-digital face-to-face consultations, though participants may prefer face-to-face contact (Mirzaei and Kashian, 2020). When it comes to stress management training, Apolinário-Hagen and colleagues also found that participants prefer face-to-face formats against digital formats without face-to-face contact (Apolinário-Hagen et al., 2020; Apolinário-Hagen et al., 2017). However, there has been no direct comparison between Group-iSMT via videoconferences and Individual-iSMT that includes written feedback by e-coaches to explore the expected advantages of these two digital stress management training approaches.

### 1.2 Objectives

The overall aim of the present study was to evaluate an established on-site group stress management training program – ‘Gelassen und sicher im Stress’ (‘calm and safe under stress’) – which we adapted and delivered via online videoconferences (Group-iSMT) and then compared to a well-proven internet-based stress management training course that included e-coach guidance (Individual-iSMT; ‘GET.ON Stress’). Specific study objectives were (1) to compare the feasibility of Group-iSMT and Individual-iSMT; and (2) to compare the potential effectiveness of each approach to generate realistic effect estimates to guide a larger, randomized controlled effectiveness trial.

## 2 Methods

### 2.1 Study design

We conducted a parallel-group, randomized controlled pilot trial with 1:1 randomization of participants to two study conditions. Outcome assessments took place immediately before (T1) and after the training period of seven weeks (T2). The primary outcome was self-perceived stress at T2. Secondary outcomes were depressive symptoms, as well as training adherence, satisfaction, and experience, including perceived benefits of each training condition. All study procedures were in full compliance with the Declaration of Helsinki for Ethical Principles for Medical Research Involving Human Subjects. The study also was approved by the ethics committee at Leuphana University of Lueneburg (202103-04-Lehr) and registered with the German Clinical Trials Register (DRKS00024965). Reporting of the present trial follows CONSORT guidelines for randomized pilot and feasibility trials (Eldridge et al., 2016).

### 2.2 Participants and their recruitment

Applicants for this trial had to fulfill the following inclusion criteria: (a) be employed, (b) have access to the internet, (c) be able to attend Group-iSMT sessions at set times, (d) give their informed consent to participate, and (e) complete the baseline assessment (T1). Applicants who reported that they were receiving psychotherapy for any kind of mental health problem were excluded. A statutory health insurance fund (Pronova BKK) supported the recruitment of participants by advertising the study via its occupational health promotion app. In Germany, statutory health insurance funds are obliged to support companies in workplace health promotion regardless of whether workers are clients of the respective health insurance fund. The insurance fund was not otherwise involved in conducting the study or analyzing results.

Recruitment took place between May and December 2021 via an open access website and was not restricted to members of the insurance fund. The website contained information on the content and available evidence of the training programs offered. After registration at the website, applicants received online access to the baseline questionnaire and a consent form including information about the study conditions. Subjects who filled the questionnaire and gave informed consent were included and randomized. We employed a restricted randomization procedure with block size set at two. The study coordinator (LB) generated the random allocation sequence. A student assistant checked whether applicants met the inclusion criteria. To ensure allocation concealment, an independent researcher who was not involved in recruitment or enrolment allocated participants to the two study arms. The independent researcher informed the study coordinator about the randomization result. The blinding of participants or trial personnel was not possible. Participants allocated to the Individual-iSMT study arm received immediate access to the training. Participants allocated to the Group-iSMT arm had to wait until enough participants registered to start a new training group. Seven weeks after their training began, all participants received an e-mail which included a link to the online post-assessment questionnaire (T2). After they completed their online, post-assessment written questionnaire, participants were invited to participate in a 30-min, semi-structured, person-to-person interview.

### 2.3 Internet-based stress management training for groups (group-iSMT)

The Group-iSMT program – “Gelassen und sicher im Stress “[Calm and safe under stress] (Kaluza, 1998, 2015) – is grounded in cognitive-behavioral techniques. Since its development, it has become very influential in health promotion practice and is considered standard stress management training in German-speaking countries. The program has four major modules, each incorporating different strategies for coping with stress: progressive muscle relaxation; problem-solving techniques; cognitive restructuring of dysfunctional attitudes; and enhancement of pleasant activities. The program includes exercises to be done within the whole group, in pairs, or by individuals on their own. A stress management trainer led each session, in accordance with the training manual (Kaluza, 2015). The trainers in the present trial were psychotherapists who had participated in a five-day train-the-trainer program, held by the developer of the ‘Calm and safe under stress’ training concept.

The course structure was adapted for videoconferencing as follows: first, participants took part in seven training sessions, each lasting 90 min and held once per week (Table 1), with a maximum of eight participants in each group. Second, at the end of each session, the participants were allotted 15 min of extra time in the virtual meeting room without the trainer being present to allow them to share informally among themselves. Third, a Master’s degree-level psychologist assisted each session to provide technical support. Fourth, all participants received a printed workbook before their first training session, which included work sheets with short explanations of all the exercises offered in the course. In addition, the participants received an e-mail reminder before each subsequent training session, which included both a link to the upcoming videoconference session and its agenda.

**Table 1:** Content of the Group-iSMT program.

Day/Session^a^	Intervention content
1	Introduction to the training schedule and getting to know each other Psychoeducation on stress and coping competencies
2	Progressive muscle relaxation I – introduction and guided practice^b^ Cognitive restructuring I – introduction to dysfunctional thinking
3	Cognitive restructuring II – coping with dysfunctional patterns of thinking Problem-solving I – self-evaluating the impact of different problems on stress
4	Progressive muscle relaxation II – extending guided practice Problem-solving II – developing an initial problem-solving plan
5	Problem-solving III – self-evaluating and adapting the problem-solving plan Enhancing pleasant activities I
6	Enhancing pleasant activities II Cognitive restructuring III – coping with dysfunctional attitudes
7	Developing a personal health promoting project for future

^aEach training session lasted 90 min. For each group, all training took place within seven weeks; bparticipants were instructed to practice the relaxation technique regularly on their own between the training days. On training days 2 and 3, participants could share their experiences with the technique and ask for any needed assistance.^

### 2.4 Internet-based stress management training for the individual (Individual-iSMT)

The Individual-iSMT program “GET.ON Stress” (Heber et al., 2013; Heber et al., 2016) was designed to enhance two strategies of stress coping: problem solving and emotion regulation (Table 2). Training consists of seven sequential sessions that participants should work on following a weekly schedule. Participants must finish each session before starting the next. Each training session consists of general information; interactive exercises; fictional training participants – so called personas – who represent different stressed employee groups; quizzes; audio and video files; and downloadable work sheets. In addition, at the end of sessions 2 through 6, users can choose to attain extra information and perform short exercises about the following common stress-related topics: time management, rumination and worrying, psychological detachment from work, sleep hygiene, sleeping habit rhythm and regularity, nutrition and exercise, organization of breaks during work, and social support. A detailed description of the exercises used in each training session can be found in the study protocol of the first study of this iSMT (Heber et al., 2013). In addition to training program content, participants received written feedback from an e-coach on their exercises after each training session, all feedback provided in accordance with the training manual. The e-coach in this trial was a Master’s degree-level psychologist. The e-coach averaged 30 min per feedback. In addition, the e-coach sent reminders to participants any time they failed to complete a training module within seven days. All communication between the participant and e-coach took place in a secured, web-based, open-source platform, located at Leuphana University of Lueneburg. A point-by-point description of both training programs, based on the template for intervention description and replication (TIDieR) (Hoffmann et al., 2014), can be viewed in the Supplementary material 1.

**Table 2:** Content of the Individual-iSMT program.

Session^a^	Intervention content
1	Psychoeducation on stress and coping competencies Enhancing pleasant activities
2	Problem-solving I – identifying and differentiating solvable and unsolvable problems; developing an initial problem-solving plan Information and exercises on additional topics, which users can self-select^b^
3	Problem solving II – self-evaluating the problem-solving plan; adapting or developing a new problem-solving plan Information and exercises on additional topics, which users can self-select^b^
4	Emotion regulation I – progressive muscle relaxation Information and exercises on additional topics, which users can self-select^b^
5	Emotion regulation II – acceptance and tolerance of (negative) emotions Information and exercises on additional topics, which users can self-select^b^
6	Emotion regulation III – effective self-support in times of stress Information and exercises on additional topics, which users can self-select^b^
7	Developing a stress-coping plan for future

^aEach session lasted approximately 45 to 90 min; bOptional exercises covered the topics of time management, rumination and worrying, psychological detachment from work, sleep hygiene, the rhythm and regularity of sleeping habits, nutrition and exercise, organization of breaks during work, and social support.^

### 2.5 Outcomes

Adherence: For the Group-iSMT program, the trainer checked and recorded the attendance of the allocated participants during each group session. For participants in the Individual-iSMT program, the online platform automatically recorded the number of completed training sessions.

Satisfaction: The eight-item Client Satisfaction Questionnaire was used, adapted for internet interventions (CSQ-I) (Boß et al., 2016) with response options of 1 = does not apply to me; 2 = minimally applies to me; 3 = moderately applies to me; and 4 = totally applies to me (summation score range: 8–32).

Training preferences: To assess preferences before and after the training, we asked participants which training format they would prefer if they could choose freely. We also asked about their knowledge of both training formats before and after training.

Training experiences: We conducted semi-structured interviews with participants from each training condition to explore (a) format-specific benefits, (b) barriers against and facilitators for attending the training sessions, (c) whether participants were able to practice exercises in their everyday lives, and (d) suggestions for improving the training received. The interview guide is provided as Supplementary material 2. The interviews lasted up to 30 min each.

Health outcomes: The primary outcome measure of training effectiveness was the Perceived Stress Scale – 10 (PSS) (Cohen et al., 1983), which consists of ten items, each having 5-point Likert scale response options of 0 = never; 1 = almost never; 2 = sometimes; 3 = fairly often; and 4 = very often (summation score range 0–40). In this trial, the participants were asked to rate all items related to the past week. To assess negative consequences of stress, we used the short form of the German adaptation of the Center for Epidemiological Studies Depression Scale (CES-D) (Hautzinger et al., 2012; Radloff, 1977), which consists of 15 items, each having 5-point Likert scale response options of 0 = rarely; 1 = sometimes; 2 = fairly often; and 3 = mostly, referring to the past week (sum score range: 0–45). A score of 18 is the threshold at or above which at least subclinical depression is suspected (Lehr et al., 2008).

### 2.6 Analysis

To compare the feasibility of the two training programs from the participants’ perspectives, we report descriptive statistics for (a) satisfaction, (b) training adherence, and (c) preference for and (d) knowledge about both training formats before and after training. In addition, we narratively present responses to open-ended questions used during the online assessment and interviews. Qualitative data were extracted using the thematic analysis approach (Braun and Clarke, 2006). To compare the two training formats’ effectiveness for health outcomes, we examined pre-to-post changes within each condition separately via repeated analysis of variance, restricting analysis to participants with a complete set of outcome data. We present means and standard deviations for the primary and secondary outcomes, as well as effect sizes – reported as Cohen’s d values – for each study condition. To explore potential differences between the two conditions, we calculated d by subtracting the mean of Group-iSMT at T2 from the mean of Individual-iSMT at T2, and dividing this by the pooled standard deviation of the T1 scores. To explore health effects at an individual level, we counted the number of participants below the cut-off value for subclinical depression in each study condition.

### 2.7 Sample size calculation

One objective of this trial was to pilot a comparison of two different stress management interventions that have already been shown to be effective. A margin of non-inferiority of d = 0.29 on the primary outcome was considered the maximum acceptable difference between the two training formats at T2. This threshold corresponds to 2 points on the Perceived Stress Scale, expecting a standard deviation of 6.3 from normative data (Klein et al., 2016). We were further guided by minimum sample sizes recommended for feasibility studies, which in the case of an anticipated small inter-group difference is *n* = 20 per study arm (Whitehead et al., 2016). Due to an unknown dropout rate with the new videoconference format, we sought to recruit 30 subjects per study arm, for a total of *N* = 60 participants.

## 3 Results

### 3.1 Participants

**Figure 1:**
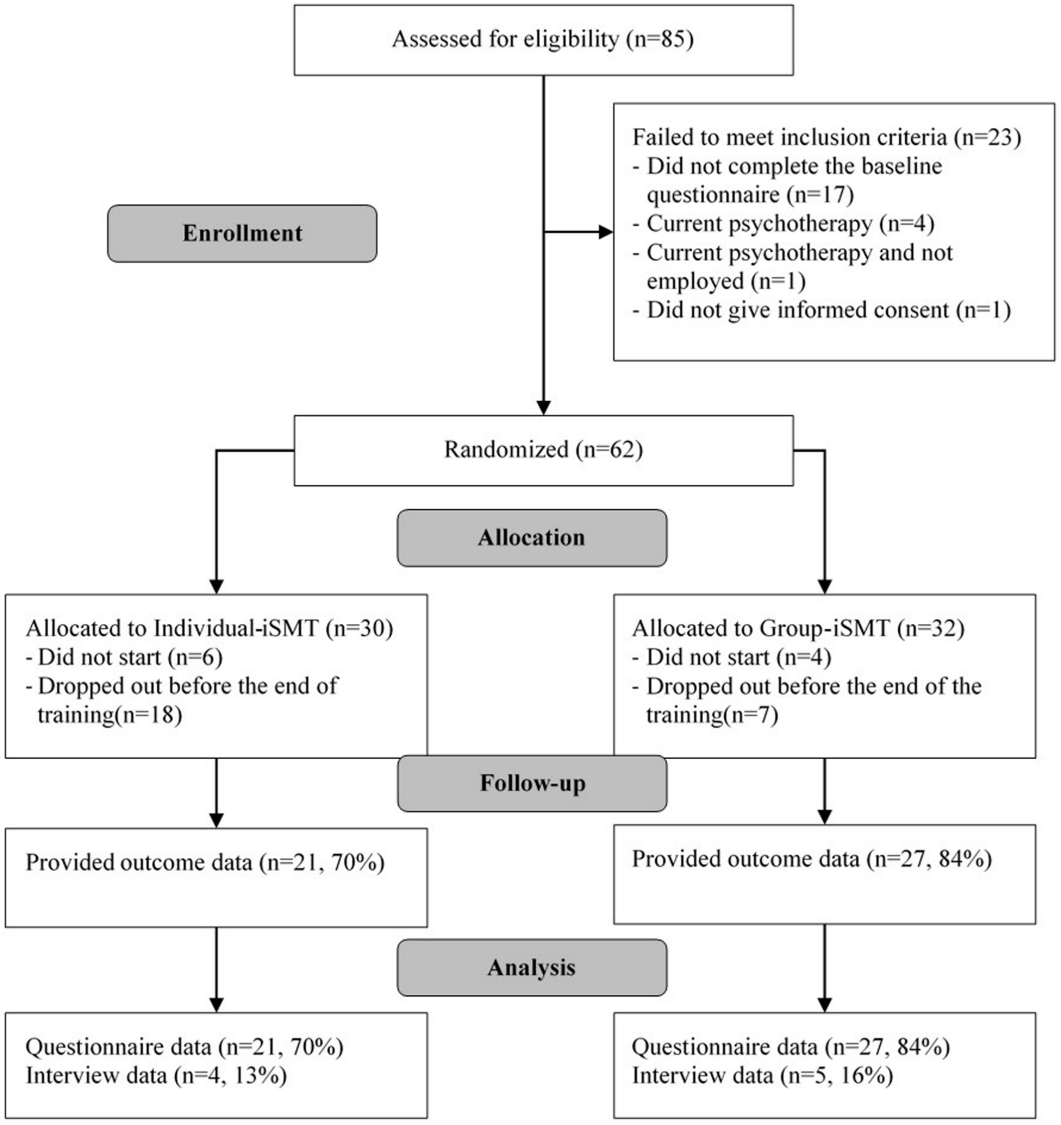
Flow of study participants.

Overall, 85 applicants registered for the study, among whom 62 ultimately gave their informed consent, completed the baseline questionnaire, and were randomized (T1; Figure 1). Most of the participants were women (49, 79%) and the average age was 44.7 (SD = 10.2; range: 23–62) (Table 3). The majority had a permanent employment contract and reported exceeding contractual working hours. Roughly one third indicated that work-related stressors were not balanced by rewards. Overall, 25 (40%) had previously participated in some form of group stress management training, while 22 (35%) had used a digital SMT format in the past. Before randomization, almost half of the participants reported a preference for an Individual-iSMT approach (29; 47%), about a quarter a Group-iSMT approach (16; 26%), and the final quarter no preference (17; 27%). Out of the 32 participants in the Group-iSMT arm, six (22%) were acquainted with at least some of their other group members before the training sessions started.

**Table 3:** Participant characteristics at baseline.

	Group-iSMT (*N* = 32)		Individual-iSMT (*N* = 30)	
	*N*	%	*N*	%
Social-economic characteristics				
Age (mean, SD)	45.8	11.0	43.7	9.4
Females	25	78.1	24	80.0
Married/partnership	17	53.1	18	60.0
Higher education	21	65.6	23	76.7
Permanently employed	25	78.1	23	76.7
Effort-reward-imbalance	11	34.3	9	30.0
Contractual working hours (mean, SD)	33,3	11.4	36.0	7.8
Actual working hours (mean, SD)	38.9	11.4	42.7	10.0
Mental health services use				
Previous digital or analogous group-based training	12	37.5	13	43.3
Previous digital single-user training	10	31.3	12	40.0
Currently receiving treatment from a general practitioner	0	0.0	2	6.7
Currently using psychopharmaceuticals	3	9.4	3	10.0
Training knowledge				
Good knowledge about Group-iSMT^a^	7	21.9	6	20.0
Good knowledge about Individual-iSMT^b^	5	15.6	9	30.0
Training preferences				
Preference for Group-iSMT	8	25.0	8	26.7
Preference for Individual-iSMT	15	46.9	14	46.7
No preference for either format	9	28.1	8	26.7

^aNumber of subjects responding ‘yes’ to the item ‘I have a clear idea of how live online group stress management training works’; bnumber of subjects responding ‘yes’ to the item ‘I have a clear idea of how an individual online training for stress management with e-coaching support works’.^

### 3.2 Adherence

**Figure 2:**
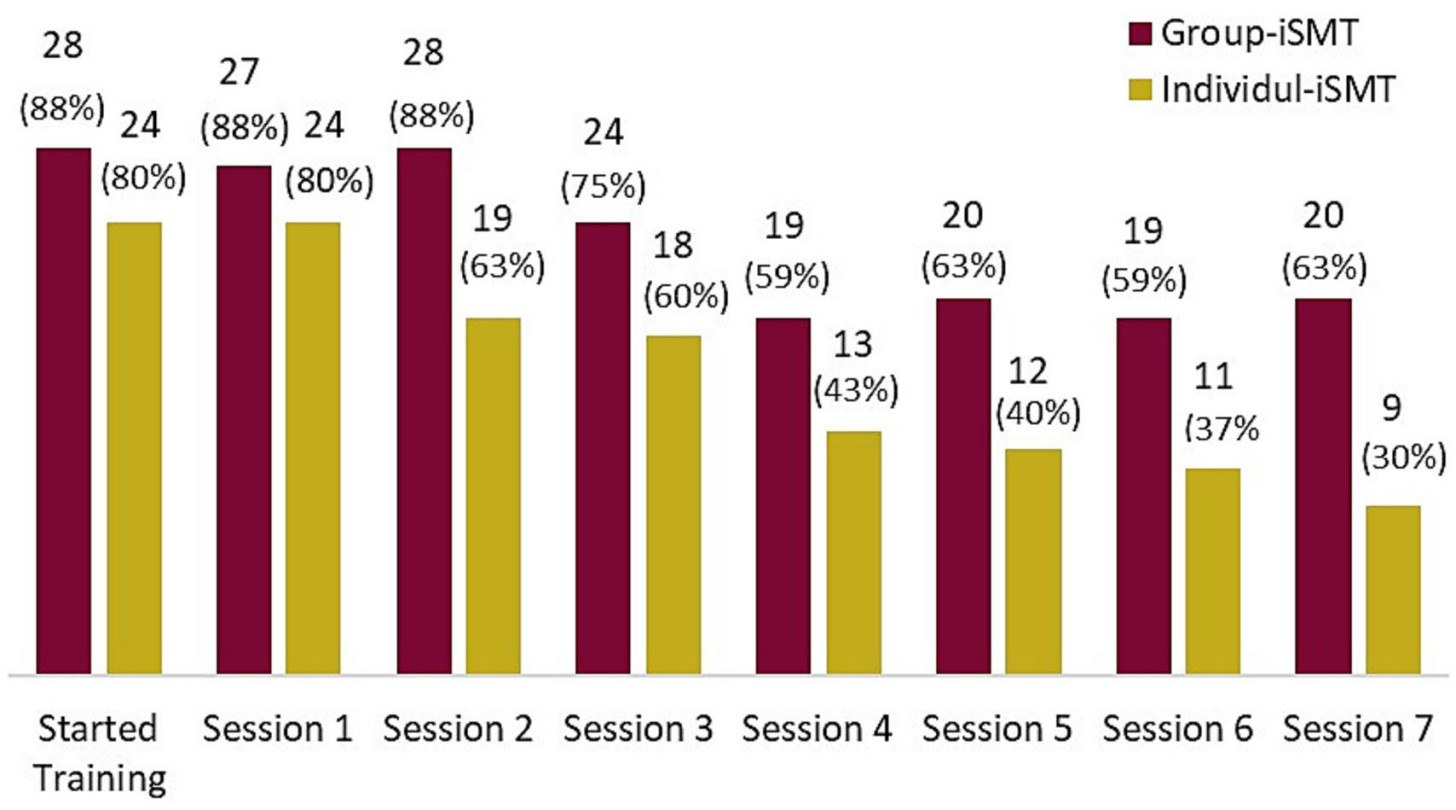
Numbers and percentages of participants in each training format completing the training sessions.

In the Group-iSMT program, four subjects terminated their participation before the first training session (Figure 1), with two reporting time constraints, one never reporting back, and another logging out during the first session while the trainer was giving technical introductions. None of these four took part in the post-assessment survey. In the Individual-iSMT program, six participants never logged in for training and none of these six responded to an e-mail reminder. On average, the adherence rate in Group-iSMT participants was 70% (M = 4.9 sessions; SD = 2.4), versus 50% among Individual-iSMT participants (M = 3.5 sessions; SD = 2.8). As shown in Figure 2, a majority of 20 Group-iSMT participants (63%) attended the last training session, compared to just nine (30%) Individual-iSMT participants.

### 3.3 Satisfaction with training received

The mean total CSQ-I score was 26.9 (SD = 4.9) among Group-iSMT and 26.5 (SD = 4.6) among Individual-iSMT participants. In the Group-iSMT group, 23 participants (85%) versus 17 (85%) in the Individual-iSMT group said they would recommend the training they received to friends if they needed help. Most of the subjects in both the Group-iSMT (25, 93%) and Individual-iSMT (18, 90%) groups reported that they could make use of what they had learned in the training program in their everyday life. The number of Group-iSMT participants with a preference for the group training format increased from 8/32 (25%) at T1 to 18/27 (67%) at T2. Likewise, among Individual-iSMT participants, the number of participants reporting a preference for the individual format increased from 14/30 (47%) to 15/20 (75%).

### 3.4 Comparative beneficial characteristics of the training formats

Asking participants about beneficial characteristics of the Group-iSMT over the Individual-iSMT format, participants highlighted their perceived value of the social support provided by other members of their training group:

“The exchange with others was very valuable. It was also good to realize that others feel the same way you do. The group was also very friendly and homogeneous, despite the different professional profiles and characters. I would want to participate in a group again.” (F_71; Group-iSMT)

“Before the training, I thought that individual online training would do me good, because in my job I do a lot of seminars with several people and I had the wish to reflect on my own and get individual support. The experience with this training has changed that and the exchanges between and composition of group members was very helpful. My fears that I might not feel comfortable or that I might worry too much about what others think were not confirmed.” (F_73; Group-iSMT)

“In the long run, real, interpersonal communication is indispensable in coaching and in a group. The real physically-experienced resonance and feedback loops are very important for me to integrate new experiences into my personal development and into my everyday behaviors, to be able to realign myself. I missed that very much during the online training.” (F_30; Individual-iSMT)

Beyond that, participants reported a higher level of commitment to attend group training sessions and perform exercises in the group:

“Easier to motivate yourself if participation is mandatory at fixed times” (F_35; Individual- iSMT)

“In the live sessions, I had to engage in exercises that I probably would not have worked on or would have worked on less intensively during an individual e-coaching session. The group dynamics motivated me (contrary to my expectations) and I was able to learn from the contributions and experiences of the other participants.” (F_32; Group-iSMT)

However, the participants also recognized some advantages in training alone. Many of the participants appreciated the fact that Individual-iSMT makes it possible to work on the trainings sessions in a flexible way in terms of time:

“Individual time allocation, no fixed day; also the state of mind, whether one just ‘feels like’ training on a day and wants to deal with it (e.g., after a long day at work or on a hot summer day) could have been chosen individually” (F_01; Group-iSMT)

“The individual time allocation suited me personally the most. In addition, the structure through the media changes was almost like being with a real trainer and the interaction with feedback very practical.” (F_23; Individual-iSMT)

Beyond flexibility, participants perceived the Individual-iSMT as allowing more space for working on individual problems or targets and appreciated the individual coaching:

“I noticed in the online group that everyone deals with the topic of stress individually and there was little that was new to me. With the individual coaching, I could have built more specifically on my previous experience.” (F_14; Group-iSMT)

Others reported a clear preference for Individual-iSMT due to their rather introverted personality:

“With my reserved personality, I don't really come into my own in small groups; it takes me too long to adjust to the situation. I have also found that the person leading the training has a significant influence on me.” (F_24; Group-iSMT)

“I am someone who likes to work on this alone, at my own pace. I am very introverted and I love to be able to work on things in writing and at my pace. So I was very happy that I was selected for the individual training and e-coaching.” (F_80; Individual-iSMT)

One participant summarized the benefits of each training format as follows:

“I didn't have a clear preference beforehand and was able to see positive and negative sides to both methods. With live online group training, you would have had contact with others, would not have felt alone with your problem, and could have asked individual questions. On the other hand, you would have had to talk about your problems in front of unknown people and use a fixed time per week. The possibility of spreading the exercises over several days with online training suited me. A fixed date would have stressed me out more. In addition, I am a visual learner and can better understand and retain material by reading and answering questions in writing.” (F_46; Individual-iSMT)

### 3.5 Reported difficulties with training received

Among Group-iSMT participants, 14 (52%) found it difficult to attend all the sessions. Nine (33%) reported that they sometimes felt uncomfortable making their own verbal contributions during the training sessions, and three (11%) said that the reason for this was that they knew some of their group members from their work before training commenced. Seven (26%) reported technical problems prior or during the group sessions. During the interviews, participants reported that “(…) In part, however, the appointments were somewhat full in terms of content, so that in the end there were sometimes time problems.” (F_24; Group-iSMT) noted that the amount of content per session was appropriate, but “intensive” (F_43; Group-iSMT). In addition, the participants found it difficult to practice what they had learned in their full days between the sessions:

“Due to time constraints, it was difficult to practice between the training sessions; but in the course of the week I have already tried one or the other.” (F_43 F_27; Group-iSMT)

“It was quite a lot of input, so even between appointments there wasn't time or opportunity (because certain situations just didn't arise within seven days) to try out what we learned in everyday life.” (F_54 F_27; Group-iSMT)

Some Individual-iSMT participants reported selective difficulties during the training with specific exercises: “The exercises were sometimes not easy to do.” (F_16; Individual-iSMT) and “I had a little trouble with the 6-step plan, but of course I could have written an email to the coach” (F_75; Individual-iSMT). Others reported difficulties with the presentation of content – “I found it to be a lot of text and not interactive enough.” (F_67; Individual-iSMT) – and expressed a wish for additional non-digital features:

“It would have been nice to have something in hand (printout, booklet or postcard for mnemonics, etc.) to be able to look something up without having to log in; for instance, regardless of training, I put a postcard with helpful sayings on the wall next to the toilet to remind me of helpful thoughts.” (F_67; Individual-iSMT)

### 3.6 Health-related effectiveness

**Figure 3:**
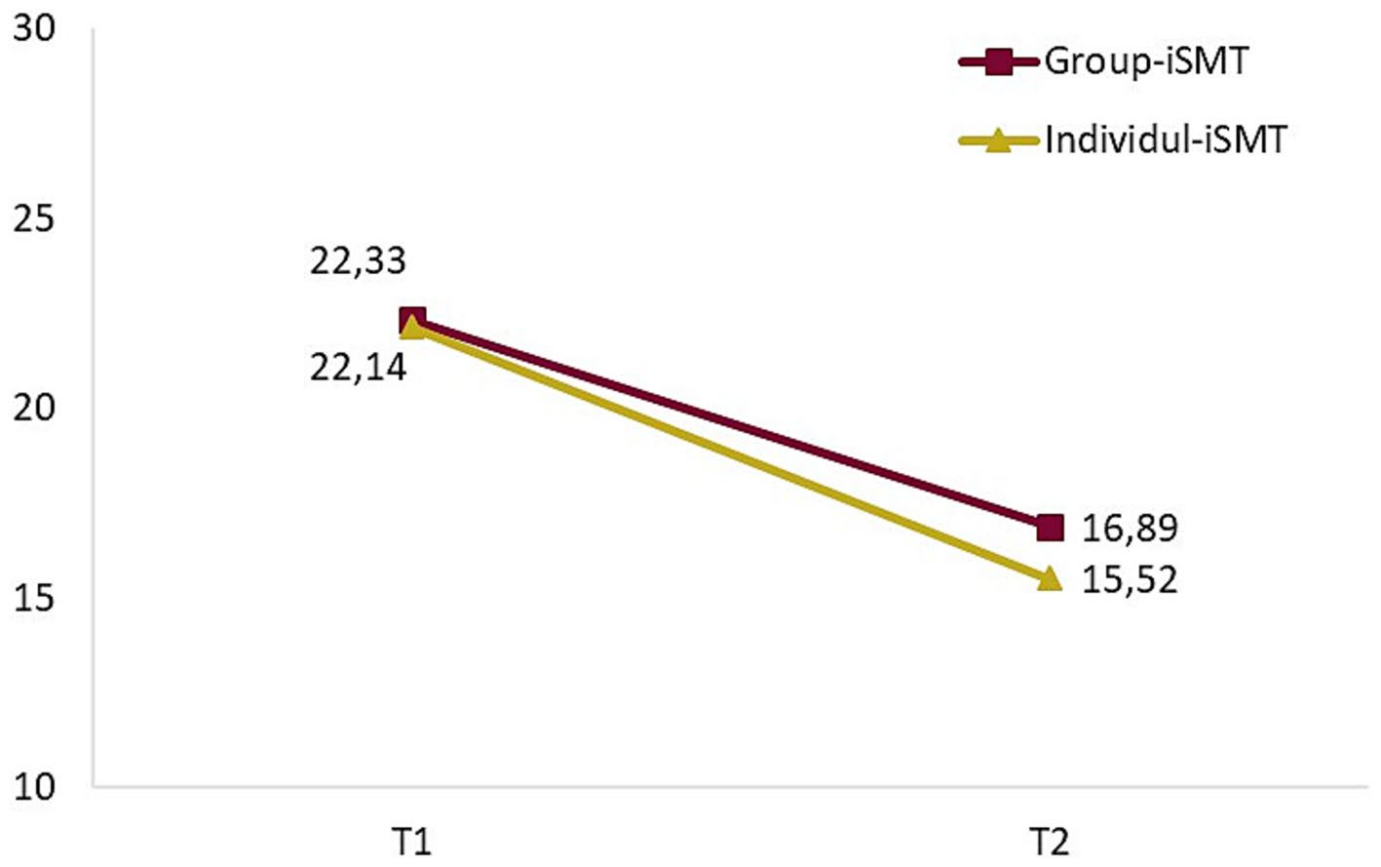
Course of perceived stress in participants with the two training conditions.

Participants in both study conditions reported statistically-significant reductions in stress (Group-iSMT: F_26,1_ = 33.53, *p* < 0.001; Individual-iSMT: F_20,1_ = 21.99, *p* < 0.001). In the Group-iSMT group, stress decreased by 5.44 points (24.4%) and in the Individual-iSMT group by 6.62 points (29.9%; Figure 3). On average, the two conditions differed in stress score by 1.37 points at T2, in favor of Individual-iSMT, which corresponds to a Cohen’s d = 0.25 (95% confidence interval: −0.32 to 0.83). As expected, this difference was not statistically significant (*p* = 0.579). Sensitivity analysis with missing values imputed using baseline observations carried forward (Supplementary material 3) revealed slightly smaller pre-to-post reductions in both conditions (Group-iSMT: 4.59 points; Individual-iSMT: 4.63 points). At an individual level, the number of participants with a depression score below the pre-defined cut-off value of 18 points increased from 16 (59%) at T1 to 21 (78%) at T2 in Group-iSMT and from 15 (71%) to 17 (81%) in Individual-iSMT participants (Table 4).

**Table 4:** Descriptive outcome data from Group-iSMT (*N* = 27) and Individual-iSMT (*N* = 21) cases with complete datasets.

Outcome	T1		T2		d^1^	95%-CI
	Mean	SD	Mean	SD		
PSS						
Group-iSMT	22.33	6.39	16.89	5.29	0.9	0.4–1.5
Individual-iSMT	22.14	4.64	15.52	5.56	1.3	0.6–2.0
CES-D						
Group-iSMT	15.11	8.90	11.07	8.53	0.5	−0.1 – 1.0
Individual-iSMT	14.55	6.77	9.35	6.39	0.8	0.2–1.4
CSQ-I						
Group-iSMT	-	-	26.89	5.16	-	-
Individual-iSMT	-	-	26.50	4.64	-	-

T1, baseline assessment; T2, post-assessment 8 weeks after randomization; PSS, Perceived Stress Scale; CES-D, Center for Epidemiological Studies Depression Scale; CSQ-I, Client Satisfaction with Internet Interventions; ^1^Cohen’s d = (Mean_pre_ – Mean_post_)/pooled SD.

## 4 Discussion

### 4.1 Principal findings

The main aim of the present study was to investigate the feasibility of delivering established stress management training, which was originally developed for on-site group settings, via videoconferencing. Results from our mixed-methods evaluations suggest that conducting SMT online in small-sized groups (five to nine members), led by a qualified trainer and a person for technical support, is both feasible and effective. With regard to satisfaction with the training, and effects on stress and depressive symptoms, no meaningful or significant differences were observed relative to a well-investigated internet-based SMT for individual format. While adherence to the group format was higher, specific advantages and shortcomings for each SMT format emerged.

Participant satisfaction with both formats was very similar to previous studies on internet-based interventions (Boß et al., 2016; Heber et al., 2016). Initially, almost half of the study participants preferred the individual format, while a quarter preferred the group format. Through the lens of self-determination theory (Ryan and Deci, 2000), this might be explained by a predominant need for autonomy over relatedness. The individual training format offered more autonomy to participants, whereas the group format provided a higher degree of relatedness. However, after the training program, the majority of participants said they would choose the same training format which they actually received, if they could choose freely. The increase in preference seemed greater for the group format and may reflect that the need for relatedness increases after experiencing positive social relationships. Similarly, the vast majority of participants (85% in both groups) said they would recommend the intervention format they experienced themselves to a friend. Mere exposure to a training format may explain this general increase in preference (Zajonc, 2001). Short informational videos could be a pragmatic way to utilize this effect and increase acceptance of digital SMT in routine occupational care (Baumeister et al., 2015; Ebert et al., 2015).

The qualitative interviews brought deeper insights into the characteristics and specific advantages of each training format. First, Group-iSMT participants highlighted that they could share experiences with one another and benefit from the effective coping strategies of others. Thus, participants seemed to value the opportunity of the group format for social learning, which may strengthen their belief in being capable to cope with stressors effectively (Bandura, 1997). An important pre-condition for such positive experiences was willingness to provide personal self-disclosure in front of a group of people and a trainer. On the other hand, participating together with a colleague who was known beforehand seemed to reduce participants’ comfort with self-disclosure. The skills of the trainer to create a safe and supportive atmosphere, facilitating constructive exchanges and integrating participants with different personality traits, appears to be another important pre-condition. These findings are in line with meta-analysis evidence that group cohesion influences treatment effects in medium-sized groups (Burlingame et al., 2018).

Second, the most important advantage of the Individual-iSMT format was its flexibility, especially in terms of time. This confirms meta-analysis findings among women in the general population (Verhoeks et al., 2019), as well as results from another study conducted in an occupational setting (Carolan and de Visser, 2018).

Third, individuals prone to introversion seemed to prefer Individual-iSMT because they worry about group cohesion. However, our results also show that such preferences can change depending on the experiences participants have. In general, this suggests that greater attention should be paid to personality traits. For example, openness to experiences has been documented to predict participants’ intention to use digital SMT (Apolinário-Hagen et al., 2020) and agreeableness found to enhance the effects of Individual-iSMT (Ebert et al., 2021).

Fourth, participants in the Individual-iSMT group valued the personalized feedback provided by an e-coach. They felt that this individual guidance granted more space for them to work more intensively on personal problems and goals. Meta-analysis evidence shows that personal guidance enhances adherence (Zarski et al., 2016) and strengthens program effects on stress reduction (Phillips et al., 2019). However, in accordance with previous findings on training preferences (Apolinário-Hagen et al., 2020), some participants would have liked to have had some live contact with the e-coach via telephone or video calls, as well. These results fit findings from a systematic review, which showed that program participants often perceive a lack of sociability using individual e-health interventions and that many wish to have more personal contact (Verhoeks et al., 2019).

Fifth, those who received group training felt obligated to the other members to attend the training sessions. Attendance in training sessions is considered an important indicator of adherence to web-based interventions (Beintner et al., 2019). Adherence to the Individual-iSMT program in the present trial (50% intervention completer) was less than what has been reported for previously-published studies on the same intervention (70%; Zarski et al., 2016). Comparing adherence rates for Group-iSMT programs is difficult, however, because research on both digital and traditional group formats is very scarce and attendance rarely reported (Miguel et al., 2023). One systematic review of interventions for healthcare professionals identified slightly higher average attendance rates for traditional group training programs of similar length delivered on-site compared to the Group-iSMT program offered in the present trial (Kunzler et al., 2020). In the present trial, Group-iSMT participants attended an average of 1.4 more sessions than Individual-iSMT participants, indicating that training in a group setting might increase adherence to the training, relative to training on one’s own. However, there are multiple possible explanations for this result. First, the training process differed between the two training formats. In the Individual-iSMT program, participants could only start the next session after they had completed the prior session, whereas Group-iSMT participants could attend later sessions even when they had missed one or more previous sessions. Second, the higher attendance rate in Group-iSMT participants might be associated not only with the group setting, but also with the fixed time schedule for the online sessions. Participants reported that both fixed appointments and feeling a sense of obligation to other group members motivated them to attend the sessions.

To summarize, the advantages and limitations of group and individual iSMT identified by our study subjects are consistent with a previous comprehensive description of 20 characteristics of digital and traditional group training (Lehr et al., 2016). That said, the present research helps to focus on those characteristics perceived as most important by participants’ themselves.

Examining health outcomes, stress reduction from 25 to 30%, relative to baseline, was reported by both groups. The pre-to-post effect of d = 0.9 that we identified for Group-iSMT corresponds to the effect reported for the original intervention when offered on-site (d = 0.7) (Kaluza, 1998). The pre-to-post effect of d = 1.3 for our Individual-iSMT program was likewise similar to that published earlier for this intervention (d = 1.5) (Heber et al., 2016). In groups with low to moderate mental distress like the participants in the present study, the effects we observed can be considered practically meaningful improvement (Bauer-Staeb et al., 2021). However, differences in observed reductions in perceived stress between the two formats were neither statistically significant nor practically meaningful (Boß et al., 2021).

### 4.2 Study strengths and limitations

One major strength of the present study is that the newly-adapted group SMT was compared to an evidence-based internet-delivered SMT for individuals that had been previously found effective in numerous published trials (Ebert et al., 2021; Ebert et al., 2016; Heber et al., 2016; Nixon et al., 2021; Nixon et al., 2022). This contrasts with prior studies, which have been criticized for using either inactive or weak active comparison conditions (Phillips et al., 2019). A second strength was employing a mixed-methods evaluation strategy that provided broader perspectives relative to adopting either a qualitative or a quantitative analytical approach alone. Moreover, this trial provides a promising example of how traditional group training concepts delivered on-site could be adapted for video conferencing.

Several study limitations should nonetheless be considered. First, results associated with each format are difficult to explain, as they differed in multiple important features: (a) group vs. single-user setting, (b) fixed versus flexible session times, and (c) synchronous contact with the trainer during sessions versus written feedback afterwards. Accordingly, the tendency toward greater adherence to iSMT in groups cannot be explained by a single factor. Second, due to the different characteristics of the training formats, we operationalized adherence using a single metric that could be assessed in both formats (i.e., number of sessions completed). Therefore, the results on adherence are limited to this metric. Third, by giving informed consent, participants indicated that they were, in principle, willing to undertake either training format. Therefore, employees with strong preferences for or reservations against one or both formats were likely underrepresented in this study. Furthermore, the participants explicitly appreciated the personal guidance included in both formats which is in line with findings from previous trials with the same individual iSMT intervention showing the personal guidance increases adherence (Zarski et al., 2016). Both factors might have contribute to the relatively high adherence found for both training formats in the present study. The reported advantages and shortcomings also may reflect only a proportion of the various program features that must be considered. Representative samples are needed to identify reasons for strong format-specific preferences. Fourth, as participation in the study was voluntary, it is assumed that participants have a high intrinsic readiness to take part in stress management training. Therefore, the results regarding adherence, satisfaction and stress reduction may only apply to employees who participate voluntarily and without direct or indirect pressure from their employer or other third parties. Finally, although results from our qualitative evaluation provided fruitful insights, they must not be generalized. They, nevertheless, might spur the generation of hypotheses that warrant being tested in further studies. Similarly, although we found no evidence of differential efficacy between the two formats in this feasibility study, this may be explained by a lack of statistical power. Larger, adequately powered non-inferiority or equivalence trials are needed to draw stronger conclusions. The present results provide valuable information that could guide sample size calculation for future definitive trials.

### 4.3 Conclusion

The present study results suggest that a stress management training program originally developed for on-site group settings may be accepted by employees offered such training via videoconferencing and that such an approach may effectively reduce stress. Participants in both digital formats – group iSMT and individual iSMT with e-coaching – benefited to a comparable and practically-meaningful extent. Moreover, whereas participants especially appreciated the peer support of the group format, they also valued the superior flexibility of individual training sessions. With both iSMT formats, participants emphasized the importance of personal contact with either a coach or helpful peers. If the non-inferiority of videoconferencing group formats can be confirmed in larger, more definitive trials, the intervention-participant fit perspective may become even more important. The characteristics of internet-based group and individual SMT that our study subjects identified should be used to develop shared-decision consultation guidelines to support stressed employees when choosing the format most appropriate for them. Furthermore, our findings might also support public health campaigns to increase to overall use of internet-based stress management training.

## Data Availability

The raw data supporting the conclusions of this article will be made available by the authors, without undue reservation. On request, the materials of the two interventions can be made available for replication studies by DL.
