# Supplementary material for: Feasibility of online group stress management training compared to web-based individual training for employees—a randomized pilot study
Source: Front Psychol. 2025 Apr 24;16:1524285. doi: 10.3389/fpsyg.2025.1524285 (PMC12058778; doi:10.3389/fpsyg.2025.1524285)
Supplement: Supplementary file 1 [file Table_1.docx]

**Supplementary material 3.** **Sensitivity analysis**: Descriptive outcomes scores for Group-iSMT (N=32) and Individual-iSMT (N=30) participants with missing values imputed as baseline observations carried forward

|  | T1 |  |  | T2 |  |  |  |  |
| --- | --- | --- | --- | --- | --- | --- | --- | --- |
| Outcome | Mean | SD |  | Mean | SD |  | d^1^ | 95%-CI |
| PSS |  |  |  |  |  |  |  |  |
| Group-iSMT | 22.47 | 6.00 |  | 17.88 | 5.52 |  | 0.8 | 0.3 – 1.3 |
| Individual-iSMT | 22.20 | 5.06 |  | 17.57 | 6.49 |  | 0.8 | 0.3 – 1.3 |
| CES-D |  |  |  |  |  |  |  |  |
| Group-iSMT | 15.44 | 8.78 |  | 12.03 | 8.73 |  | 0.4 | -0.1 – 0.9 |
| Individual-iSMT | 15.83 | 7.39 |  | 12.37 | 8.17 |  | 0.4 | -0.1 – 1.0 |
| Notes: T1 = baseline assessment; T2 = post-assessment 8 weeks after randomization; PSS = Perceived Stress Scale; CES-D = Center for Epidemiological Studies Depression Scale; ^1^ Cohen’s d = Mean_pre_ – Mean_post_ / pooled SD) | | | | | | | | |
