# Supplementary material for: Feasibility of online group stress management training compared to web-based individual training for employees—a randomized pilot study
Source: Front Psychol. 2025 Apr 24;16:1524285. doi: 10.3389/fpsyg.2025.1524285 (PMC12058778; doi:10.3389/fpsyg.2025.1524285)
Supplement: Supplementary file 3 [file Data_Sheet_2.docx]

**Supplementary material 2. Interview Guide for participants in the iSMTgroup [iSMTindividual]**

Thank you for being willing to share your training experience with me. It has been X weeks since the last training session.

1. Thinking about the process from signing up for the training to the first appointment [first login to the training]...What was that like for you? Was there a waiting period or were you able to get started right away?
2. How did you like the training? What exactly did you particularly like/what maybe not so much?
3. Have you participated in similar training or workshops in the past (whether digital or on-site)?
4. If you have experience with on-site workshops/training:
   1. Was there anything that went more smoothly/better for you in the online group training [web-based training] than usually on-site?
   2. Was there anything that did not work out so well in the online group training [web-based training] from your perspective/what was difficult compared to an on-site workshop?
5. How well did you manage to try out one or more things from the training in your everyday life, even between the group sessions [web-based sessions]?
6. Is there anything from the training you would like to continue in the next few weeks or that you would like to learn more about or try out?
7. If you could ask for training on stress management, what would it look like? Online or on-site/ within a group of peers or by yourself? How many appointments/sessions? How long would the appointments last? Who would you most like to train with in the case of a group training?
8. Is there anything else you would like to share with me that we have not talked about yet?
